# Lipedematous Scalp: Presentation of a Rare Dermatological Condition

**DOI:** 10.7759/cureus.63504

**Published:** 2024-06-30

**Authors:** Jing Huang, Casey Hudson, Olivia Connolly, David B Kessler

**Affiliations:** 1 Medicine, Nova Southeastern University Dr. Kiran C. Patel College of Osteopathic Medicine, Fort Lauderdale, USA; 2 Neuroscience, Binghamton University, Binghamton, USA; 3 Biology, Binghamton University, Binghamton, USA; 4 Dermatology, New York Institute of Technology College of Osteopathic Medicine, New York, USA

**Keywords:** scalp dysesthesia, increased scalp thickness, hyperlipidemia, lipedematous alopecia, lipedematous scalp

## Abstract

Lipedematous scalp (LS) and lipedematous alopecia (LA) are uncommon dermatological conditions characterized by lipid accumulation within scalp tissue, leading to a thickened and boggy scalp. While the exact cause remains elusive, these conditions are believed to be on a spectrum of the same underlying disease process. LS/LA patients can experience dysesthesia of the scalp, but LA is associated with additional hair growth abnormalities. The pathogenesis remains poorly understood, with some cases suggesting a link to hormone leptin dysregulation and/or hyperlipidemia. We present a 73-year-old African American female with a medical history of hypertension, hyperlipidemia, and partial thyroidectomy who presented to the clinic with a two-week history of an itchy, burning 'rash' on the scalp. Physical examination showed normal hair density, but palpation revealed scalp edema and diffuse bogginess. While blood tests were mostly normal, she had an elevated antinuclear antibodies (ANA) titer (1:160). A punch biopsy revealed lichenification, but subsequent non-contrast magnetic resonance angiography (MRA) showed increased scalp fat tissue measuring up to 11 mm, confirming the diagnosis of LS. The patient was reassured that this finding was benign; however, she continued to experience dysesthesia. Our patient experienced minimal relief with topical steroids, leading to the consideration of intralesional steroid injections. The case highlights the importance of recognizing and managing LS as a distinct dermatological entity that requires further research to elucidate underlying mechanisms and establish standardized treatment protocols for this condition.

## Introduction

Lipedematous scalp (LS) and lipedematous alopecia (LA) are rare and underreported dermatological conditions characterized by the excessive accumulation of lipids within the scalp tissue, resulting in a diffusely thickened and boggy scalp [1,2]. Though the etiology of LS and LA remains unknown, recent literature suggests that they represent a spectrum of the same underlying disease [1]. Patients with LS are typically asymptomatic but can present with paresthesia, pruritus, and pain [2], in contrast with LA, where there are additional abnormalities related to hair growth [1]. The pathogenesis of both LS and LA is not well understood; however, some cases have reported an association with possible hormone leptin dysregulation and/or hyperlipidemia [3]. The vertex and occiput are the most commonly affected scalp regions, but the disease can progress to involve the entire scalp [4]. This condition primarily affects women, with around 90% of reported cases occurring in this demographic [1]. Individuals of African American or Egyptian descent appear to be disproportionately affected, comprising roughly half of all cases [1]. However, the condition is not exclusive to these populations as cases have also been documented in Caucasian patients of both genders [5]. The median age of onset is 48 years old [1,4,6]. Diagnosis involves a combination of clinical examination, punch/incisional biopsy, and imaging tests to show increased subcutaneous adipose tissue leading to increased scalp thickness [1]. Normal scalp thickness is on average 5.8±0.12 mm but patients with LS or LA have an average scalp thickness of 11.4 mm [3]. This case report provides details on the presentation, diagnosis, and management of a patient with LS, highlighting the rarity and challenges associated with this condition.

## Case presentation

**Figure 1:**
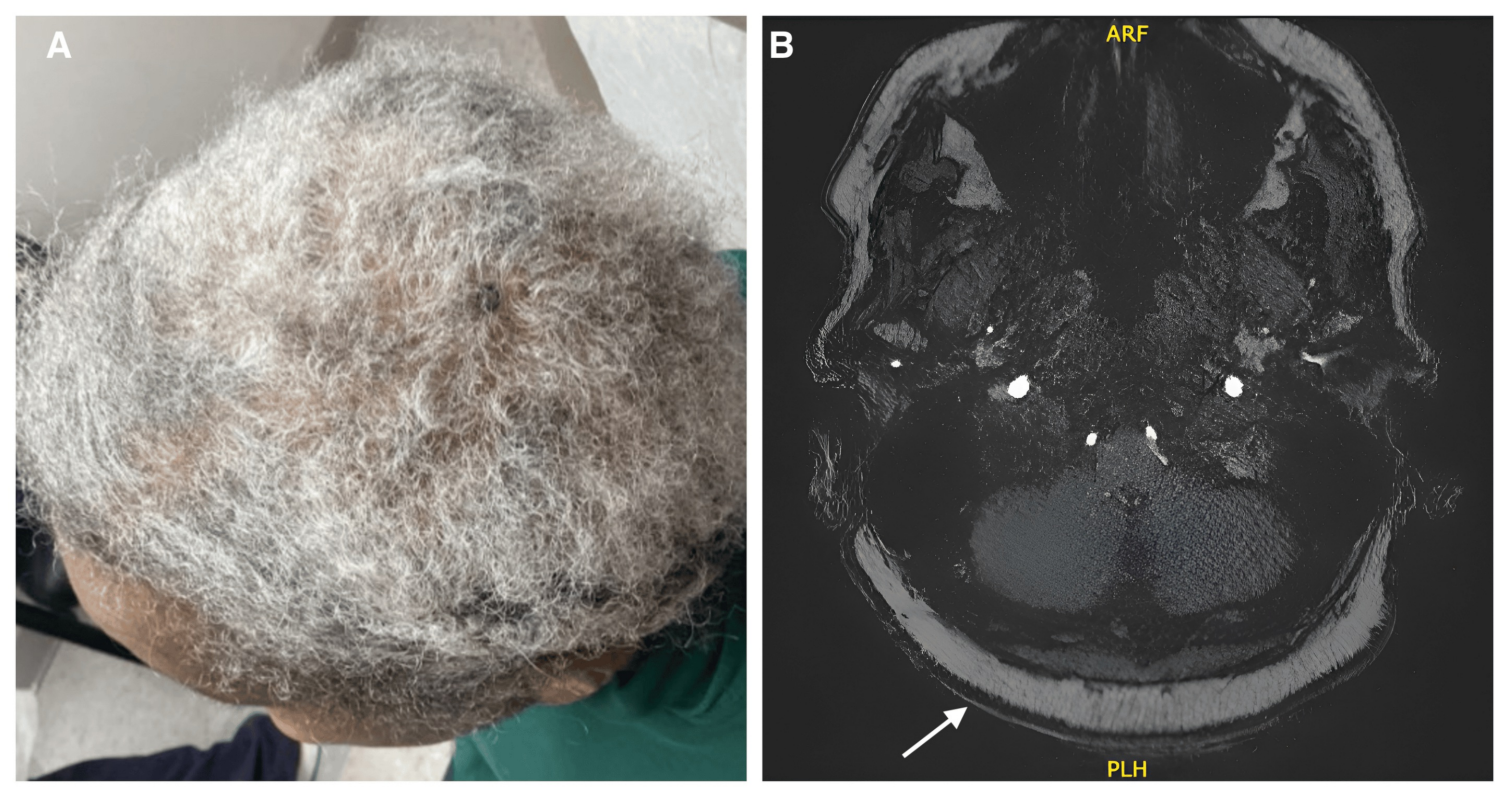
(A) Thickened and boggy scalp with normal hair distribution; (B) Non-contrast MRA brain showing prominent scalp fat tissue measuring up to 11 mm in thickness MRA: magnetic resonance angiography

A 73-year-old African American female with a history of hypertension, hyperlipidemia, and partial thyroidectomy presented to the clinic with a chief complaint of a "rash" on the scalp. The rash had been present for two weeks, accompanied by itching and a burning sensation. She denied any changes to personal care products or new medications in the interim. Her medical conditions are managed with low-dose aspirin, amlodipine, atorvastatin, and metoprolol. Scalp examination revealed normal hair density with no apparent abnormalities, but palpation indicated scalp edema and diffuse bogginess. The patient was prescribed mometasone 0.1% topical solution for her scalp dysesthesia, and additional blood tests were ordered to investigate the rash, including thyroid function tests, connective tissue disease (CTD) panel, antinuclear antibody (ANA), Sjögren's syndrome antibody (Ab), and anti-SSA/SSB antibodies. Complete blood count and thyroid function tests were within normal limits, but the patient had an elevated ANA (1:160). At the two-week follow-up, the burning sensation had slightly improved with topical steroid use, but it was also accompanied by intermittent itching, mainly on the left central parietal scalp. A punch biopsy of the left central parietal scalp revealed features of lichenification, including the involvement of a few hair follicles with spongiosis. No signs of alopecia were identified, consistent with the physical exam findings of normal hair growth. In a separate evaluation for vertigo by her primary physician, a non-contrast magnetic resonance angiography (MRA) of the brain revealed prominent fat tissue located on the scalp measuring up to 11 mm in thickness (Figure 1B). Based on the imaging and physical exam findings, a diagnosis of LS was made. The patient was reassured of the benign nature of this scalp condition. Follow-up visits and scalp examinations continued to show a boggy and edematous scalp. She is currently being followed in the clinic for symptomatic management of her scalp dysesthesia. Since topical steroids have provided minimal symptomatic relief, the patient agreed to a trial course of intralesional steroid injections.

## Discussion

LS was first described by Cornbleet in 1935, followed by LA by Coskey et al. in 1961 [1, 4]. Since its first appearance in literature, there have been about 100 cases of LS/LA reported to date. The pathophysiology of LS and LA remains unclear, but the hallmark of these two diseases is a thickening of the subcutaneous tissue, approximately double that of a healthy scalp [7]. The mechanism behind hair loss in LA is postulated to be due to the increased thickness of the subcutaneous tissue that leads to a disruption of the blood supply to the hair bulb, resulting in follicular atrophy [3]. Due to the predominance of LS/LA affecting the female population, it is hypothesized that hormone dysregulation of leptin may play a role in the pathophysiology by causing increased accumulation of subcutaneous fat [1]. Other authors have reported cases of associated metabolic derangements such as hyperlipidemia in some patients with LA [3]. Although our patient has a history of hyperlipidemia, it is currently well controlled with medications and her lipid panel was within normal limits, making hyperlipidemia a less likely underlying cause of her LS. While no reliable link exists between LS/LA and other systemic diseases or genetic mutations, blood work can help rule out underlying organic causes.

Kilinc et al. suggested a possible link between LA and autoimmune diseases such as discoid lupus erythematosus (DLE), highlighting a case where an ANA-positive patient with LA presented with similar histopathological features to late-phase DLE, such as hyperkeratosis, follicular plugging, and fibrosis [2]. While our patient's blood work was positive for ANA, she presented with no hair growth abnormalities and a biopsy negative for histopathological features of LA. This prompts us to believe that the positive ANA may have been an unrelated finding. There is currently not enough evidence to support the association between LA and autoimmune disease, but it is certainly a field that needs further exploration. We recommended that she follow up with a rheumatologist for further evaluation given her lab results.

Currently, there are no established treatment guidelines for LS and LA. Management for LS primarily focuses on relieving associated scalp dysesthesia, if present. A previously reported case of spontaneous regression of LS supports its generally benign nature [8]. For LA, previously documented treatments include intralesional triamcinolone injections and mycophenolate mofetil (MMF) [1,9]. Cabrera et al. demonstrated successful treatment of LA in a patient after a 10-month course of MMF, hypothesized to be due to its inhibition of perifollicular fibrosis observed in the histopathology of the hair follicles in LA [9]. Given our patient's increased scalp thickness evident on both MRA and physical exam, she was diagnosed with LS. With the absence of hair growth abnormalities and a biopsy unremarkable for LA features, we opted for symptomatic treatment.

## Conclusions

This case highlights the importance of recognizing and managing LS as a distinct dermatological condition. For patients presenting with complaints of dysesthesia and lack of hair growth abnormalities, a thorough physical examination including scalp palpation is crucial for revealing the characteristic boggy and edematous feel of the scalp. This initial assessment can then be followed by imaging to definitively measure scalp tissue thickness. In cases where hair growth abnormalities are also present, a punch biopsy may provide valuable insights into the thickened scalp tissue. Histopathological examination can reveal characteristic findings of lipedematous alopecia, such as hyperkeratosis, follicular plugging, and fibrosis which are crucial for guiding appropriate treatment decisions. Currently, there are no standardized treatment guidelines for the management of LS/LA. Further research is crucial to elucidate the underlying mechanisms of LS and establish standardized diagnostic criteria and treatment protocols.
